# Supplementary material for: Comparative Analysis of Chromatin-Delivered Biomarkers in the Monitoring of Sepsis and Septic Shock: A Pilot Study
Source: Int J Mol Sci. 2021 Sep 14;22(18):9935. doi: 10.3390/ijms22189935 (PMC8465401; doi:10.3390/ijms22189935)
Supplement: Supplementary file 1 [file ijms-22-09935-s001.zip › ijms-1262258-supplementary.pdf]

**Correlation** Spearman r		A	B	C	D	E	F	G	H	I	J	K	L	M	N	O	P	Q	R
		Nucl. kit 1	CitrH3	Nucl. kit 2	HMGB1	MBI	ICU LOS	HOSP LOS	SOFA	SAP	MAP	RF	ALPH	BIL	GLUC1	TTPA1	Fibrin1	DD1	NtProBNP1
1	Nucl. kit 1	1.000	0.006	0.455	0.248	0.012	0.226	0.055	-0.328	0.316	0.347	0.632	-0.900	0.054	-0.207	0.812	0.812	0.086	0.000
2	CitrH3	0.006	1.000	0.770	0.030	-0.598	0.378	0.348	0.712	-0.729	-0.711	0.337	-0.100	0.559	0.188	0.406	0.667	0.771	-0.536
3	Nucl. kit 2	0.455	0.770	1.000	0.152	-0.720	0.421	0.311	0.557	-0.347	-0.292	0.607	-0.600	0.577	-0.061	0.551	0.812	0.771	-0.429
4	HMGB1	0.248	0.030	0.152	1.000	0.195	0.854	0.701	0.105	0.280	-0.067	0.448	-0.100	-0.234	-0.657	-0.522	0.174	0.886	-0.786
5	MBI	0.012	-0.598	-0.720	0.195	1.000	-0.126	-0.113	-0.757	0.532	0.382	-0.198	0.667	-0.360	-0.208	-0.493	-0.145	-0.371	0.036
6	ICU LOS	0.226	0.378	0.421	0.854	-0.126	1.000	0.929	0.405	-0.122	-0.333	0.642	-0.359	-0.109	-0.306	-0.412	0.206	0.943	-0.873
7	HOSP LOS	0.055	0.348	0.311	0.701	-0.113	0.929	1.000	0.408	-0.287	-0.327	0.661	-0.100	0.109	-0.040	-0.471	-0.029	0.886	-0.873
8	SOFA	-0.328	0.712	0.557	0.105	-0.757	0.405	0.408	1.000	-0.525	-0.503	0.188	-0.103	0.426	0.065	-0.194	-0.239	0.667	-0.599
9	SAP	0.316	-0.729	-0.347	0.280	0.532	-0.122	-0.287	-0.525	1.000	0.808	-0.129	0.100	0.000	-0.579	-0.176	-0.088	-0.314	0.036
10	MAP	0.347	-0.711	-0.292	-0.067	0.382	-0.333	-0.327	-0.503	0.808	1.000	0.049	0.100	0.432	-0.134	0.551	-0.058	-0.812	0.321
11	RF	0.632	0.337	0.607	0.448	-0.198	0.642	0.661	0.188	-0.129	0.049	1.000	-0.359	0.374	-0.086	0.708	0.708	0.577	-0.630
12	ALPH	-0.900	-0.100	-0.600	-0.100	0.667	-0.359	-0.100	-0.103	0.100	0.100	-0.359	1.000	0.400	-0.300	-0.500	0.000	0.500	-0.800
13	BIL	0.054	0.559	0.577	-0.234	-0.360	-0.109	0.109	0.426	0.000	0.432	0.374	0.400	1.000	0.209	0.353	0.221	0.205	-0.174
14	GLUC1	-0.207	0.188	-0.061	-0.657	-0.208	-0.306	-0.040	0.065	-0.579	-0.134	-0.086	-0.300	0.209	1.000	0.132	-0.441	-0.580	0.378
15	TTPA1	0.812	0.406	0.551	-0.522	-0.493	-0.412	-0.471	-0.194	-0.176	0.551	0.708	-0.500	0.353	0.132	1.000	0.603	-0.616	0.000
16	Fibrin1	0.812	0.667	0.812	0.174	-0.145	0.206	-0.029	-0.239	-0.088	-0.058	0.708	0.000	0.221	-0.441	0.603	1.000	0.462	-0.435
17	DD1	0.086	0.771	0.771	0.886	-0.371	0.943	0.886	0.667	-0.314	-0.812	0.577	0.500	0.205	-0.580	-0.616	0.462	1.000	-0.900
18	NtProBNP1	0.000	-0.536	-0.429	-0.786	0.036	-0.873	-0.873	-0.599	0.036	0.321	-0.630	-0.800	-0.174	0.378	0.000	-0.435	-0.900	1.000

**Correlation** P values		A	B	C	D	E	F	G	H	I	J	K	L	M	N	O	P	Q	R
		Nucl.kit1	CitrH3	Nucl.kit2	HMGB1	MBI	ICULOS	HOSPLOS	SOFA	SAP	MAP	RF	ALPH	BIL	GLUC1	TTPA1	Fibrin1	DD1	NtProBNP1
1	Nucl. kit 1		1.000	0.191	0.492	0.979	0.529	0.884	0.351	0.372	0.324	0.057	0.083	0.917	0.565	0.072	0.072	0.919	1.000
2	H3citr	1.000		0.013	0.946	0.073	0.280	0.323	0.025	0.021	0.025	0.343	0.950	0.206	0.600	0.433	0.156	0.103	0.236
3	Nucl. kit 2	0.191	0.013		0.682	0.023	0.226	0.379	0.099	0.324	0.411	0.069	0.350	0.186	0.872	0.272	0.072	0.103	0.354
4	HMGB1	0.492	0.946	0.682		0.587	0.003	0.028	0.773	0.432	0.857	0.198	0.950	0.623	0.044	0.300	0.750	0.033	0.048
5	MBI	0.979	0.073	0.023	0.587		0.726	0.752	0.014	0.116	0.272	0.583	0.267	0.426	0.558	0.333	0.778	0.497	0.963
6	ICU LOS	0.529	0.280	0.226	0.003	0.726		2.690e-004	0.244	0.734	0.343	0.050	0.633	0.817	0.385	0.419	0.686	0.017	0.017
7	HOSP LOS	0.884	0.323	0.379	0.028	0.752	2.690e-004		0.239	0.415	0.352	0.043	0.950	0.813	0.915	0.342	0.956	0.033	0.016
8	SOFA	0.351	0.025	0.099	0.773	0.014	0.244	0.239		0.122	0.140	0.601	0.900	0.348	0.858	0.756	0.667	0.156	0.162
9	SAP	0.372	0.021	0.324	0.432	0.116	0.734	0.415	0.122		0.006	0.722	0.950	1.000	0.083	0.747	0.864	0.564	0.952
10	MAP	0.324	0.025	0.411	0.857	0.272	0.343	0.352	0.140	0.006		0.897	0.950	0.327	0.710	0.272	0.933	0.072	0.498
11	RF	0.057	0.343	0.069	0.198	0.583	0.050	0.043	0.601	0.722	0.897		0.567	0.408	0.815	0.150	0.150	0.250	0.152
12	ALPH	0.083	0.950	0.350	0.950	0.267	0.633	0.950	0.900	0.950	0.950	0.567		0.750	0.683	1.000	1.000	1.000	0.333
13	BIL	0.917	0.206	0.186	0.623	0.426	0.817	0.813	0.348	1.000	0.327	0.408	0.750		0.647	0.500	0.700	0.767	0.750
14	GLUC1	0.565	0.600	0.872	0.044	0.558	0.385	0.915	0.858	0.083	0.710	0.815	0.683	0.647		0.811	0.400	0.239	0.406
15	TTPA1	0.072	0.433	0.272	0.300	0.333	0.419	0.342	0.756	0.747	0.272	0.150	1.000	0.500	0.811		0.211	0.300	1.000
16	Fibrin1	0.072	0.156	0.072	0.750	0.778	0.686	0.956	0.667	0.864	0.933	0.150	1.000	0.700	0.400	0.211		0.433	0.378
17	DD1	0.919	0.103	0.103	0.033	0.497	0.017	0.033	0.156	0.564	0.072	0.250	1.000	0.767	0.239	0.300	0.433		0.083
18	NtProBNP1	1.000	0.236	0.354	0.048	0.963	0.017	0.016	0.162	0.952	0.498	0.152	0.333	0.750	0.406	1.000	0.378	0.083	
